# Deep transcriptome sequencing of subgenual anterior cingulate cortex reveals cross-diagnostic and diagnosis-specific RNA expression changes in major psychiatric disorders

**DOI:** 10.1038/s41386-020-00949-5

**Published:** 2021-02-08

**Authors:** Nirmala Akula, Stefano Marenco, Kory Johnson, Ningping Feng, Kevin Zhu, Anton Schulmann, Winston Corona, Xueying Jiang, Joanna Cross, Bryce England, Aparna Nathan, Sevilla Detera-Wadleigh, Qing Xu, Pavan K. Auluck, Kwangmi An, Robin Kramer, Jose Apud, Brent T. Harris, C. Harker Rhodes, Barbara K. Lipska, Francis J. McMahon

**Affiliations:** 1 Human Genetics Branch, National Institute of Mental Health Intramural Research Program, NIH, DHHS, Bethesda, MD USA; 2 Human Brain Collection Core, National Institute of Mental Health Intramural Research Program, NIH, DHHS, Bethesda, MD USA; 3 Bioinformatics Section, National Institute of Neurological Disorders and Stroke, NIH, DHHS, Bethesda, MD USA; 4 Georgetown Brain Bank, Histopathology and Tissue Shared Resource, Georgetown University Medical Center, Washington, DC USA

**Keywords:** RNA sequencing, Gene expression

## Abstract

Despite strong evidence of heritability and growing discovery of genetic markers for major mental illness, little is known about how gene expression in the brain differs across psychiatric diagnoses, or how known genetic risk factors shape these differences. Here we investigate expressed genes and gene transcripts in postmortem subgenual anterior cingulate cortex (sgACC), a key component of limbic circuits linked to mental illness. RNA obtained postmortem from 200 donors diagnosed with bipolar disorder, schizophrenia, major depression, or no psychiatric disorder was deeply sequenced to quantify expression of over 85,000 gene transcripts, many of which were rare. Case–control comparisons detected modest expression differences that were correlated across disorders. Case–case comparisons revealed greater expression differences, with some transcripts showing opposing patterns of expression between diagnostic groups, relative to controls. The ~250 rare transcripts that were differentially-expressed in one or more disorder groups were enriched for genes involved in synapse formation, cell junctions, and heterotrimeric G-protein complexes. Common genetic variants were associated with transcript expression (eQTL) or relative abundance of alternatively spliced transcripts (sQTL). Common genetic variants previously associated with disease risk were especially enriched for sQTLs, which together accounted for disproportionate fractions of diagnosis-specific heritability. Genetic risk factors that shape the brain transcriptome may contribute to diagnostic differences between broad classes of mental illness.

## Introduction

Genome-wide association studies (GWAS) have revealed that major psychiatric disorders share many common genetic variants that exert small effects on risk [1, 2]. Similarly, gene expression studies in postmortem brain tissue have found that expression changes in mental illnesses tend to be small and correlated across diagnoses. One large postmortem study [3] found small changes in gene expression in schizophrenia (SCZ) versus controls that seemed to reflect small differences in frequencies of alleles associated with SCZ by GWAS [4]. A meta-analysis of postmortem microarray expression data found that many differentially-expressed (DE) genes were shared across psychiatric disorders such as SCZ, autism, and bipolar disorder (BD) [5]. A large follow-up study that used RNA sequencing to more fully characterize expression of genes and transcripts—collectively known as the transcriptome—confirmed substantial overlap in DE genes in SCZ, BD, and autism spectrum disorder [6].

If genetic risk factors and expression changes in brain are as similar across different mental illnesses as these studies suggest, then how do diagnostic differences in onset, symptoms, and treatment response arise? Diagnostically distinct genetic risk factors may account for some of these differences (e.g., genetic correlations as high as the 70% reported between SCZ and BD [1] are still consistent with up to 50% of risk alleles differing between disorders). It is also possible that published gene expression studies have overestimated similarities of gene expression changes across disorders. Even the largest sample sizes have been underpowered to detect subtle gene expression differences [3], and those differences that have been detected postmortem may not be representative of dynamic expression changes across the lifespan. Most studies have focused on prefrontal cortex, even though other brain regions have been implicated in various mental illnesses [7]. Case–control study designs limit opportunities to detect true differences between case groups, and few published postmortem studies have done direct, case–case comparisons between disorders. Moreover, few if any human postmortem studies have used methods capable of revealing the full transcriptional complexity of the brain, where numerous gene isoforms are generated by alternative splicing and other mechanisms. How much of the apparent resemblance in gene expression reported among clinically dissimilar mental illnesses by earlier studies can be explained by these limitations?

To address these questions, we performed deep sequencing of the brain transcriptome followed by comparisons of both gene and isoform expression in postmortem brain tissue from 200 individuals diagnosed with BD, SCZ, major depressive disorder (MDD), or no known psychiatric illness. Total RNA was sequenced at a very high average depth of ~270 million reads per sample, generating high-quality expression data for ~21K genes and ~85K transcripts. We believe these data represent the most complete sampling of the human brain transcriptome published to date.

Tissue was dissected from subgenual anterior cingulate cortex (sgACC), a key component of limbic circuits involved in reward, impulse control, and emotion regulation [8–10]. The sgACC, which lies near the genu of the corpus callosum, has been repeatedly implicated in mood disorders by neuroimaging, neuropathology, and antidepressant treatment response studies in both humans and animals (reviewed in [9]). While one previous study characterized gene expression in total ACC obtained from people with BD [11], to our knowledge no previous studies have examined the sgACC transcriptome in both mood and psychotic disorders.

Our results demonstrate that subtle differences in gene expression observed between broad diagnostic classes of mental illness are actually underlain by more pronounced and diagnosis-specific expression changes at the transcript level, that rare transcripts are important, and that transcript-level expression is influenced by inherited genetic risk factors. We conclude that genetic risk factors shape the brain transcriptome and may contribute to diagnostic differences between broad classes of mental illness.

## Methods

A brief summary of the “Materials and Methods” follows. See Supplementary Methods for details.

### Samples and RNA-sequencing

This study was reviewed and approved by the NIMH Human Brain Collection Core Oversight Committee. Clinical information on the 200 postmortem brain samples (Controls = 61, SCZ = 46, BD = 39, and MDD = 54) is presented in Supplementary Table 1. Total RNA extracted from frozen dissections of sgACC was captured using the RiboZero protocol, followed by library preparation. Stranded, paired-end sequencing was peformed on the Illumina HiSeq 2500. We obtained an average of 270 million reads per sample, totaling ~54 billion reads (Supplementary Table 2). After quality control, reads were mapped to human genome build 38 using Hisat2 [12]. Gene and transcript counts were obtained using StringTie [12]. At least 10 read counts/sample were mapped to each of 21,228 genes and 85,295 known transcripts. Pairwise correlation in gene counts identified 15 outlier samples which were excluded from further analysis (Supplementary Fig. 1). The downstream analyses included 185 samples (55 Controls, 44 SCZ, 35 BD, and 51 MDD).

### Covariate selection and differential expression analysis

To avoid spurious differences between diagnostic groups attributable to demographic, technical, or ancestry differences across samples, we tested all 33 known demographic and technical variables and 10 genetic ancestry vectors for association with diagnosis and gene expression. Stepwise logistic regression found that only antipsychotic exposure was associated with diagnosis (Supplementary Table 3a). Linear regression found that RNA integrity number (RIN), self-reported race, and GC content were associated with one or more principal components of gene expression (Supplementary Table 3b).

Based on these results, RIN, race, and GC content were included as covariates in the differential expression analysis, while antipsychotic exposure, which is strongly associated with SCZ, was tested in post hoc analyses. DE genes and transcripts were identified using DESeq2 [13, 14] with lfcShrink option. Unless noted otherwise, results were deemed significant at an analysis-wide false-discovery rate (FDR) of 5%. Overlaps of DE genes/transcripts between this and previous studies were assessed with the hypergeometric test. The analysis pipeline is detailed in Supplementary Fig. 2.

Six genes and transcripts with RNAseq read counts >500/sample were validated using RT qPCR (Supplementary Methods). The direction of expression changes were concordant and highly correlated with the RNAseq analyses (*R*^2^ = 0.81) (Supplementary Fig. 3).

### SNP genotyping and sample verification

All samples were genotyped on Illumina genome-wide SNP arrays. Common markers (MAF > 5%) were extracted and imputed against the Haplotype Reference Consortium [15]. PLINK [16] was used to check for gender discordances. Sample identity was further verified by comparing SNP genotypes to transcribed variants called from RNA seq reads using BCFtools [17]. PLINK and R were used to calculate concordance rates between SNP genotypes and variants called from the RNAseq data. All concordance rates exceeded 99%.

### Quantitative trait loci (QTLs) associated with gene/transcript expression or relative transcript abundance

Normalized, covariate-corrected expression values from DESeq2 were combined with SNP array data to identify QTLs. Matrix eQTL [18] was used to identify SNPs within 1 Mb of a known gene/transcript that were associated with expression of that gene/transcript. We designate these as eQTLs in results that follow. MeCS [19] was used to combine eQTLs called from our sgACC data with those called independently in GTEx and CMC samples.

SNPs associated with both expression and diagnosis were identified with Summary-based Mendelian Randomization (SMR) [20] analysis of eQTLs and summary statistics for SCZ, BD, and MDD obtained from published GWAS.

SNPs associated with relative abundance of known transcripts in a gene were detected using sQTLseekeR [21]. We designate these as sQTLs in the results that follow.

### Heritability

Partitioned heritability estimates for eQTLs and sQTLs were performed with linkage disequilibrium score regression (implemented in LDSC using the standard baseline functional annotations [22, 23]), and summary statistics obtained from published GWAS of SCZ, BD, MDD, and (as a negative control) Alzheimer’s disease. See Supplementary Methods for details.

## Results

Here we present gene-level and transcript-level results, including case–control and case–case comparisons, followed by analyses of the relative contributions of eQTLs and sQTLs to disease heritability. We conclude with an assessment of the importance of rare transcripts.

### Gene-level, case–control comparisons

**Fig. 1:**
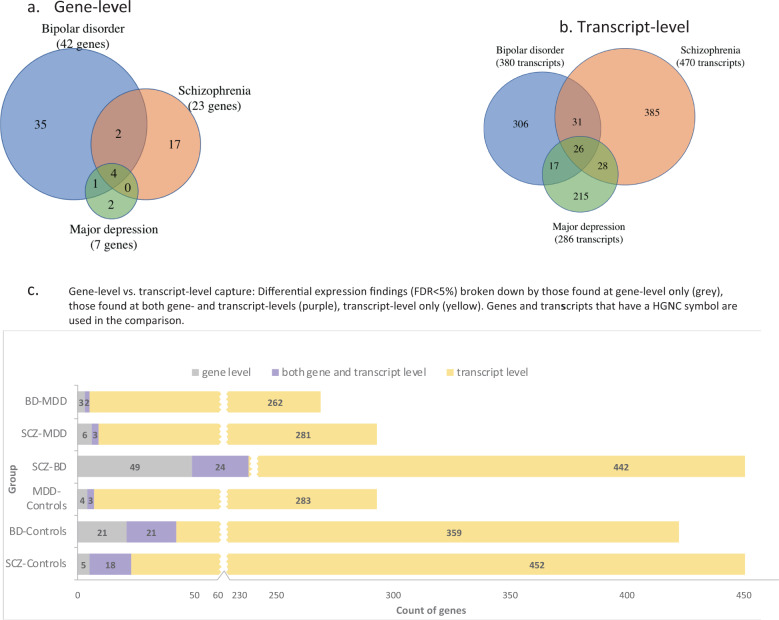
Overlap of differentially expressed genes and transcripts across disorders. Venn diagrams show counts of DE (FDR < 5%) genes (**a**) or transcripts (**b**) in case–control comparisons for all three disorders. In (**c)** differential expression findings are broken down by those found only at the gene level (gray), at both gene and transcript levels (purple), or at the transcript level only (yellow).

Differential expression between cases and controls was modest. A total of 23, 42, and 7 genes were DE at FDR < 5% in SCZ, BD and MDD, respectively. No gene displayed absolute log_2_FC values >0.5 in any diagnostic group. Seven genes were DE in multiple disorders (Fig. 1a). Genes DE in more than one diagnostic group are shown in Table 1. All DE genes with nominal *p* < 0.05 are shown in Supplementary Tables 4–6. Volcano plots for case–control comparisons are shown in Supplementary Fig. 4a.

**Table 1:** Genes differentially expressed (FDR < 5%) in >1 diagnostic group.

Diagnostic group	Number of overlap	Ensemble gene ID	Gene name	Direction of change same in all disorders	Gene description	Significance of overlap
All three groups^a^	4	ENSG00000064300	*NGFR*	Down	Nerve growth factor receptor	6.6865E−22
		ENSG00000171551	*ECEL1*	Down	Damage induced neuronal endopeptidase	
		ENSG00000179869	*ABCA13*	Down	ATP binding cassette subfamily A member 13	
		ENSG00000256861	*AC048338.1*	Down	paralog of VPS33A	
BD and SCZ	Above 4 + 2	ENSG00000172987	*HPSE2*	Down	Heparanase 2	5.17E−12
		ENSG00000175793	*SFN*	Up	Stratifin, epithelial cell marker protein	
BD and MDD	Above 4 + 1	ENSG00000146066	*HIGD2A*	Down	HIG1 hypoxia inducible domain family member 2A	6.064E−13

^a^The SCZ-MDD *p* value is 4.29E−11 but the overlapping genes are not shown because they are the same four genes indicated above in “All three groups”.

Exploratory analyses of combined diagnostic groups (SCZ and BD versus Controls; SCZ, BD, and MDD versus controls) yielded only DE genes that were already detected in individual case–control comparisons (see Supplementary Tables 4, 5 for details). This suggests the combined case groups lost signal due to heterogeneity or that the individual case–control comparisons were somehow biased by small sample size. To rule out the latter, we randomly reduced the combined case sample to approximate the size of the smallest individual diagnostic group. Comparison with controls detected only one DE gene, demonstrating that smaller samples did not paradoxically generate more DE genes.

**Fig. 2:**
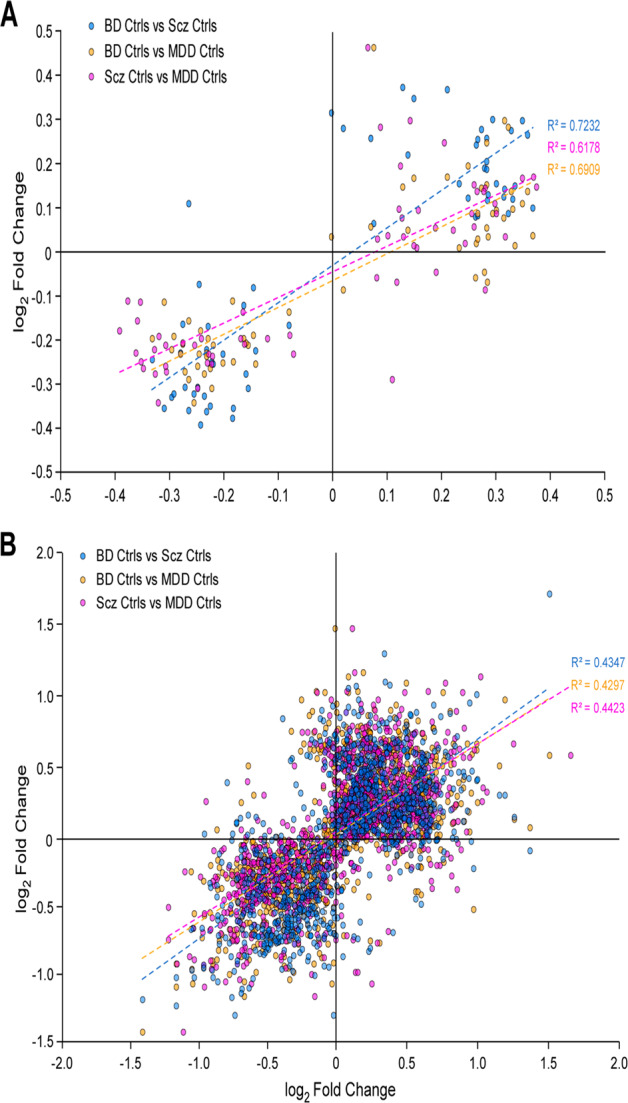
Magnitude of differential expression across disorders. Log2 fold-change values for each disorder (relative to controls) were calculated for all genes (**A**) and transcripts (**B**) that were differentially-expressed in any disorder (FDR < 5%), then plotted against log2 fold-change values for each of the other disorders. Least-squares regression lines (dotted) are shown for each pairwise comparison, along with Pearson correlation coefficients.

Among genes DE within any one diagnostic group, there was a strong positive correlation in FC values between diagnoses (linear regression *R*^2^ = 0.61–0.72) (Fig. 2A), consistent with previous studies [5, 6]. DE genes detected at FDR < 5% in sgACC significantly overlapped with previous results from total anterior cingulate cortex (ACC) [24] carried out in SCZ (hypergeometric *p* value = 2.0 × 10^−5^) or in a combined analysis of all cases (hypergeometric *p* value = 3.5 × 10^−5^). Similarly, we found significant overlap with DE genes previously reported [6] in dorsolateral prefrontal cortex (DLPFC) in SCZ (hypergeometric *p* value = 8.0 × 10^−6^) and BD (hypergeometric *p* value = 6.8 × 10^−5^) (Supplementary Tables 4, 5). Differential expression of several metallothionein genes was also detected, as reported previously [25]. Post hoc testing indicated that most DE genes could not be explained by differences in antipsychotic exposure or death by suicide (Supplementary Tables 4–9). These results demonstrate good agreement with previous studies, despite differing brain regions, samples, and methods.

### Gene-level, case–case comparisons

To interrogate differential expression between diagnostic groups, we conducted analyses in all possible case–case comparisons (BD versus SCZ, MDD versus SCZ, and MDD versus BD). These revealed a greater number of DE genes and larger FC values, on average, than the case–control comparisons (Supplementary Fig. 4b). A total of 73 genes were DE in BD versus SCZ, 9 genes were DE in MDD versus SCZ, and 5 genes were DE in MDD versus BD (Supplementary Tables 7–9).

### Gene-level eQTLs

To assess the impact of common genetic variants on gene expression, a total of 6,071,916 SNPs were tested against expression of 21,228 genes. Of these 92,081,468 SNP-gene pairs, 290,338 were associated with expression of 5780 genes at FDR < 5% (designated “eGenes,” Supplementary Table 10). Since common SNPs vary by ancestry, the analyses were repeated in samples solely from self-described Caucasians, yielding 199,421 cis-eQTLs for 4116 genes at FDR < 5%. The counts of eGenes were within expected range, given the sample size (https://gtexportal.org/home/tissueSummaryPage), and bootstrap analysis of 90% of the sample demonstrated that false-discovery rates were close to the expected 5%.

eGenes from sgACC were compared to those reported in DLPFC by Common Mind Consortium (CMC-DLPFC) [3] and those reported in anterior cingulate cortex by GTEx (GTEx-ACC) [26]. About 50% of eGenes detected in sgACC overlapped with either CMC-DLPFC or GTEx-ACC (Supplementary Fig. 5; Supplementary Table 11). A total of 2,740 eGenes were detected only in sgACC and 881 of these were not found in the largest postmortem DLPFC study [6] suggesting that they are either specific to sgACC or not sufficiently expressed in DLPFC to be detected by previous RNA-sequencing studies [6]. Consistent with this, 521 eGenes detected in sgACC were expressed at <100 reads/sample.

### Gene-level integrative analyses

To identify SNPs associated jointly with both clinical diagnosis and gene expression in brain, we integrated published GWAS with brain eQTLs using SMR, a formal test for significant joint effects [20]. To increase power, we combined our eQTL results (Caucasian-only) with GTEx-ACC and CMC-DLPFC, increasing sample size to over 800.

As reported previously [19], sample size was strongly predictive of the number of significant SMR “hits.” In SCZ, we detected 20 variants associated with diagnosis and gene expression in sgACC. This number increased to 36 in the combined sgACC and GTEx-ACC samples, and increased further to 133 when CMC-DLPFC samples were added (Supplementary Table 12). This increased power enabled detection of 69 SCZ-linked genes not found in previous studies [6, 27], including *TSHR*, which encodes the thyroid stimulating hormone receptor. In BD, 4 variants were associated with both diagnosis and gene expression in sgACC, increasing to 16 when all brain samples were considered (Supplementary Table 13). These variants were associated with expression of 19 unique genes. These results replicated 10 genes reported previously [6, 28] and detected 9 novel gene linkages, including *ORMDL3*, which regulates endoplasmic reticulum mediated calcium signaling. MDD analysis yielded 4 significant gene linkages, 2 of which are novel (Supplementary Table 14).

### Transcript-level, case–control comparisons

Transcript-level comparisons between cases and controls showed, despite a greater multiple-testing burden, 2–3 times more significant expression differences than the gene-level comparisons (Fig. 1c). This was due to a larger average expression difference at the transcript than the gene level (distributions of gene-level vs. transcript-level absolute log_2_FC values differed at *p* < 0.0001, Kolmogorov–Smirnov test). At FDR < 5% the number of DE transcripts was 470 in SCZ, 380 in BD, and 286 in MDD, a gradient rougly paralleling clinical severity (Supplementary Fig. 4c; Supplementary Tables 15–17). Over 90% of DE transcripts were predicted to be protein coding (Supplementary Table 18). Many DE transcripts were expressed at <100 reads/sample and have not to our knowledge been previously reported (Supplementary Fig. 6).

In contrast to gene-level analyses, transcript-level analyses identified substantial diversity in DE transcripts across disorders. The mean overlap of DE transcripts across disorders was 18% (Fig. 1b), with a modest correlation in FC values across diagnoses (*R*^2^ = 0.43–0.44; Fig. 2B). At least 2 different isoforms of 76 genes were DE in one or more diagnostic groups (Supplementary Fig. 7). We also detected 42 instances of alternative isoform usage, where distinct transcripts of the same gene were DE in different disorders (e.g., *GNAS* in Fig. 2C).

Our transcript-level results replicated differential expression of 22 transcripts in SCZ and two transcripts in BD previously reported by PsychENCODE [6] (Supplementary Tables 15,16). These results represent small but significant overlaps between PsychENCODE and the present study (SCZ: hypergeometric *p* value = 0.018; BD: hypergeometric *p* value = 0.039), despite methodologic differences.

### Transcript-level, case–case comparisons

To further investigate transcript-level differences between diagnoses, case–case comparisons were performed, in which each diagnostic group was contrasted with all others, as in the gene-level comparisons described above. This revealed 841 transcripts that were DE in one or more comparisons, 542 of which had not emerged from case–control comparisons alone (Supplementary Fig. 4d; Supplementary Tables 19–21).

**Fig. 3:**
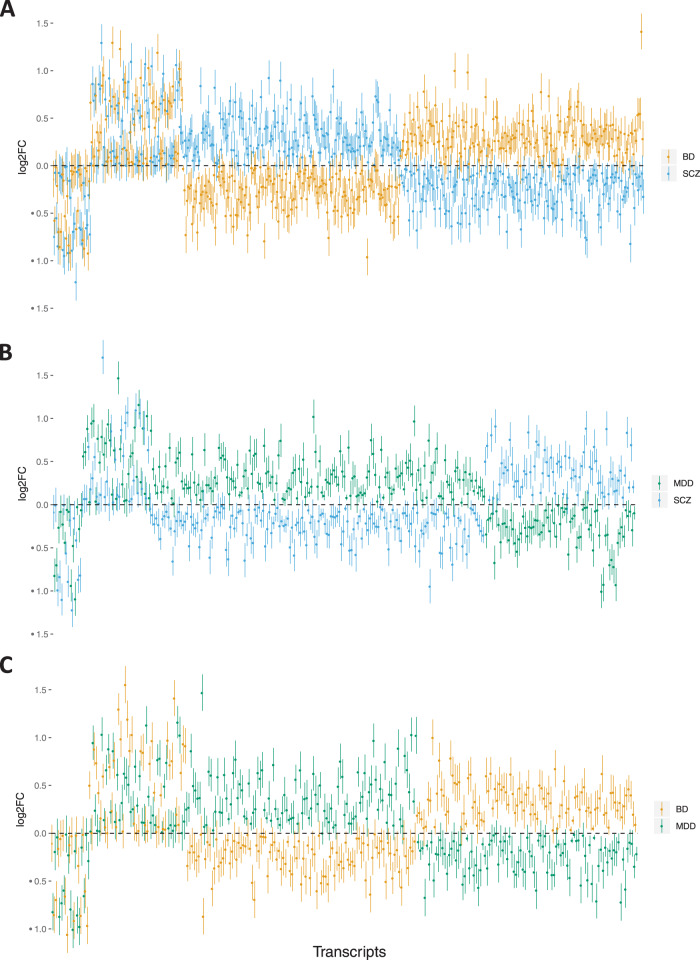
Transcript-level comparisons between case groups. Direction of expression in each case group relative to controls is shown on right (**A**, **B**, **C**), grouped by direction of change. Error bars represent standard errors.

Most of the DE transcripts in the case–case comparisons were expressed in opposite directions between diagnostic groups, relative to controls. Among all DE transcripts in the case–case contrasts, 77% (SCZ versus BD), 83% (SCZ versus MDD), and 77% (BD versus MDD) displayed this “mirror” expression pattern (Fig. 3A–C; Supplementary Tables 22–24). Reshuffling of diagnostic labels dramatically reduced the number of DE transcripts, but the proportions of DE transcripts with “mirror” expression findings were similar.

Post hoc testing indicated that most of the DE transcripts could not be explained by differences in antipsychotic exposure or death by suicide (Supplementary Tables 15–17, 19–21).

### Transcript-level eQTLs

Integration of SNPs with transcript counts detected 208,966 eQTLs affecting expression of 6008 transcripts (Supplementary Table 25). These eQTLs significantly overlapped with transcripts that were DE in SCZ (hypergeometric *p* value < 10^−5^) or BD (hypergeometric *p* value < 0.01), but not in MDD. Common alleles may thus contribute to disorder-specific differential transcript expression.

### Transcript-level integrative analyses

To quantify how much risk alleles contribute to transcript-level differences between disorders, transcript-eQTLs were integrated with summary results from recent GWAS by SMR. This revealed linkages between transcript expression and disease risk at 18 SCZ and 4 BD risk loci (Supplementary Table 26). In SCZ and BD, significantly overlapping sets of transcripts were associated with both risk allele and diagnosis (hypergeometric *p* value = 0.03 and 0.04, respectively).

### Functional enrichment of DE transcripts

Genes harboring transcripts DE at FDR < 5% in any of the case–control comparisons were significantly enriched for functions related to synapse and antigen processing (Supplementary Table 27), largely driven by the SCZ versus Ctrls comparison (Supplementary Table 28). In case–case comparisons (Supplementary Table 29), transcripts DE in SCZ versus BD were enriched for functions related to muscle or motor proteins, while SCZ versus MDD transcripts were enriched for spectrin repeats and cell membrane. No significant functional enrichment was found for the relatively smaller number of BD versus MDD transcripts.

Functional analysis of “mirror” transcripts showed significant enrichment for spectrins (Bejamini *q* < 3.12E−04). Spectrins are involved in neuronal migration and synaptic plasticity and are thought to give strength and flexibility to neurons [29, 30].

### sQTLs

In addition to changes in gene or transcript-level expression, SNPs can modify the transcriptome by driving shifts in relative abundance of transcripts within a gene [31]. These are known as splicing or sQTLs, due to their putative effect on alternative splicing [21]. In the total sample we detected 14,054 SNPs associated with relative transcript abundance within 844 genes at FDR < 5%; 9773 SNPs in 500 genes at FDR < 5% in Caucasians (Supplementary Table 30; Supplementary Fig. 8). The larger set of sQTLs meeting the nominal *p*-threshold of 0.05 (Supplementary Table 31) is used in subsequent analyses.

Consistent with their role in alternative splicing [32], most sQTLs lie near known splice sites or regions of open chromatin (Supplementary Figs. 9, 10; Supplementary Tables 32, 33) and 63% of the transcripts in genes harboring sQTLs were predicted to result from classical alternative splicing events (Supplementary Fig. 11).

Genes associated with an sQTL were significantly enriched for transcripts that were DE in any case–control (*n* = 368; OR = 2.2, hypergeometric *p* < 10^−16^) or case–case (*n* = 335; OR = 2.5, hypergeometric *p* < 10^−16^) comparison. Similar enrichments were seen across all comparisons, with odds ratios of 1.8 (SCZ versus controls) to 2.7 (BD versus MDD). “Mirror” transcripts were also enriched for sQTLs (hypergeometric *p* < 0.049) but not eQTLs. Together these results suggest that sQTLs play a disproportionate role in diagnosis-associated differential expression.

### sQTLs within GWAS loci

To test how large a role sQTLs play in disease risk, we examined all *p* < 0.05 sQTLs for evidence of association with risk for psychiatric disorders based on published GWAS [28, 33, 34]. Here we included only sQTLs that were detected in sgACC from self-reported Caucasian brain donors, since existing GWAS are mainly based on samples of European ancestry (Supplementary Methods).

Overall, about 10–25% of GWAS loci we investigated harbored at least one significant sQTL. In SCZ, of 430 known genome-wide significant GWAS SNPs, 56 were identified as an sQTL, implicating 44 genes. Of these, 15 distinct genes are linked here for the first time to changes in transcript expression within a SCZ GWAS locus. In BD, of 329 known genome-wide significant GWAS SNPs, 47 were identified as sQTLs. In MDD, 10 of 44 known GWAS loci habored sQTLs. See Supplementary Tables 34–36 for details.

### Relative contributions of eQTLs and sQTLs to disease heritability

Previous studies have demonstrated that eQTLs substantially contribute to heritability for a variety of common disorders [35], but few studies have investigated the specific contribution of sQTLs. Thus we estimated the proportion of heritability potentially explained by sQTLs, relative to eQTLs, in SCZ, BD, and MDD.

We found that sQTLs accounted for disproportionate fractions of heritability in SCZ and BD, but not MDD (Table 2). Overall, sQTLs comprised only 4% of all SNPs analyzed but explained 7–8% of heritability, a 1.7- to 2.0-fold enrichment. In contrast, sQTLs did not show any significant enrichment of Alzheimer’s disease heritability.

**Table 2:** Disorder-specific heritability attributable to common variants, SNP heritability of disorders partitioned across sQTLs and eQTLs^a^.

Diagnosis	Schizophrenia	Bipolar disorder	Major depression	Alzheimer’s disease
N SNPs	10,69,224	9,62,367	10,60,414	10,35,603
Observed h2 (se)	0.4 (0.015)	0.3 (0.018)	0.05 (0.003)	0.046 (0.009)
Proportion sQTLs	0.04	0.04	0.04	0.04
Observed sQTL h2 (se)	0.08 (0.01)	0.07 (0.01)	0.05 (0.01)	0.06 (0.04)
sQTL enrichment (se)	2.1 (0.26)	1.7 (0.31)	1.2 (0.26)	1.5 (0.90)
sQTL enrichment p	3.3E−05	0.02	0.38	0.55
Bonferroni p	9.8E−05	0.06	1	1
Proportion eQTLs	0.06	0.06	0.06	0.06
eQTL h2 (se)	0.11 (0.01)	0.08 (0.01)	0.05 (0.01)	0.12 (0.04)
eQTL enrichment (se)	1.7 (0.18)	1.3 (0.22)	0.8 (0.17)	2.0 (0.71)
eQTL enrichment p	9.00E−05	0.13	0.41	0.14
Proportion eQTLs not sQTLS	0.02	0.02	0.02	0.02
eQTL h2 (se)	0.03 (0.007)	0.02 (0.009)	0.01 (0.008)	0.08 (0.032)
eQTL enrichment (se)	1.16 (0.27)	0.81 (0.38)	0.48 (0.31)	3.33 (1.3)
eQTL enrichment p	0.54	0.62	0.10	0.05

^a^Excludes eQTLs that are also sQTLs.

### Are rare transcripts important?

We investigated the value of deep RNA-sequencing performed in the present study by comparing the numbers of known genes and transcripts identified in this study with those identified in PsychENCODE [6]. To facilitate this comparison, we recalled our data at the same threshold that was used in PsychENCODE [6] (0.1 Transcripts Per Million in 25% of samples).

The results demonstrated that high-depth RNA-sequencing performed in this study identified larger numbers of known genes and transcripts than PsychENCODE, despite a sample size only 1/10th as large. We identified 26,397 distinct genes and 109,932 transcripts, compared to 25,774 genes and 96,042 transcripts identified in PsychENCODE. In addition, 31,333 known transcripts detected in the present study were not reported by PsychENCODE [6] (Supplementary Table 37). Of these, 12,613 represent rare transcripts with mean counts <100/sample. These rare transcripts also appear to be disease relevant, since 269 were DE at FDR < 5% in either SCZ, BD, or MDD. The rare transcripts also appear to be functionally relevant, representing genes that are significantly enriched for functions related to synapse formation, cell junctions, and heterotrimeric G-protein complexes (*q* values all <5%). These results illustrate the importance of rare, DE transcripts in psychiatric disorders.

## Discussion

This study highlights transcriptome differences in genetically similar, but clinically distinct, mental illnesses. The results show that subtle differences in gene expression are actually underlain by more pronounced and diagnosis-specific changes at the transcript level. These changes are most evident in case–case comparisons, often involve alternative splicing, and are influenced by common genetic variants known to play a role in the inherited risk of mental illness.

Several brain transcriptome studies of mental illness have been published recently, some with larger samples than employed here [3, 6, 36]. This study complements those in several ways. Previous genome-wide studies in total ACC represented <50 samples [37–39] and no previous studies evaluated the sgACC, despite its importance in mood disorders [9–11]. None of the previous studies employed RNA-sequencing at a depth comparable to that used here, which enabled identification of numerous rare transcripts that were DE in particular case groups and represented genes involved in important aspects of neurobiology. Some of the genes and transcripts whose expression was associated with known risk alleles were detected only in ACC, consistent with the idea that common risk alleles may regulate expression in a tissue-specific manner [40]. We also employed case–case comparisons that revealed unexpectedly large numbers of transcripts that were DE between diagnostic groups and showed opposite directions of expression, relative to controls (“mirror transcripts”). This shows that genetically correlated psychiatric disorders can express partially contrasting brain transcriptomes that may underlie some differences in onset, symptoms, or treatment response observed between diagnoses. This finding also suggests that combining samples with different diagnoses to increase sample size may also increase heterogeneity and obscure some true signals, especially in genes harboring “mirror” transcripts.

Most transcripts whose expression was associated with genetic variants in this study are predicted to arise from classical alternative splicing mechanisms, consistent with previous findings that alternative splicing plays an important role in major psychiatric and other disorders [32, 40, 41]. Our results further demonstrate that common genetic variants associated with relative transcript abundance (sQTLs) account for disproportionate fractions of disorder-specific heritability, providing support for the proposition that alternative splicing is a primary mechanism whereby genetic variants confer risk for disease [32].

This study has several limitations. While this was one of the larger RNA-sequencing studies performed in human brain tissue to date, there were <100 individuals in each diagnostic category, limiting power to detect small expression changes. As in all human postmortem studies, diverse factors affect expression, not all of which can be measured or controlled, increasing noise and risk for bias. We addressed this by stringent quality control, careful adjustment for key covariates, and testing robustness of the major findings with alternative analysis approaches. Human postmortem studies inherently conflate expression changes that cause disease with those resulting from disease or treatment. While most DE genes and transcripts were apparently unrelated to antipsychotic drug exposure or suicide, other confounds cannot be ruled out. The integrative genomic approaches we used identify genes or transcripts whose expression is associated with both diagnosis and inherited risk alleles, which limits findings attributable to reverse causation but cannot rule out correlated non-causal events [20]. The degree to which expression levels were similar across diagnostic groups may have been influenced by use of a common control group, but this finding is quite consistent with previous large studies [5, 6] and known overlaps in genetic risk factors. A portion of the “mirror transcript” findings may reflect the fact that differential expression analysis favors genes and transcripts with low expression in one group and higher expression in another, so that a third group will tend to lie in between. Finally, sequencing of RNA derived from bulk tissue means that findings driven by differences in cellular composition cannot be fully resolved. Nevertheless, these bulk data reflect a much larger portion of the brain transcriptome than can be achieved with current single-cell technologies.

We suggest that inherited genetic risk factors shape the brain transcriptome and contribute to diagnostic differences between broad classes of mental illness. More work is needed to characterize the functions of alternative transcripts, developmental timing of alternative splicing events, potential impact of medications and other environmental exposures, and transcriptomic differences in specific brain regions or cell types.

## Funding and disclosure

This study was funded by the following two NIMH grants: ZIA-MH002810-16 and ZIC-MH002903-12. All the authors declare no competing interests. An early draft of this study has been posted on BioRxiv at https://www.biorxiv.org/content/10.1101/598649v1.

## Tissue availability

Tissue samples are available to qualified scientists upon review by the NIMH Human Brain Collection Core oversight committee. Information is available at https://www.nimh.nih.gov/research/research-conducted-at-nimh/research-areas/research-support-services/hbcc/distribution-of-resources.shtml.

## Supplementary information

Supplementary Methods

Supplementary Figures

Supplementary Tables

## Data Availability

The raw data can be downloaded from dbGAP at https://www.ncbi.nlm.nih.gov/projects/gap/cgi-bin/study.cgi?study_id=phs000979.v2.p2. The genome-wide association study (GWAS) summary results were obtained from PGC for Schizophrenia (http://www.med.unc.edu/pgc/results-and-downloads), Bipolar Disorder GWAS dataset and Major Depression GWAS dataset were obtained from PGC and UKbiobank (early access upon request). The Alzheimer’s GWAS summary results were download from IGAP (http://web.pasteur-lille.fr/en/recherche/u744/igap/igap_download.php). The Common Mind Consortium (CMC) eQTL dataset for DLPFC was downloaded via Synapse after data access agreement was approved (https://www.synapse.org). The GTEx eQTL data for anterior cingulate region (ACC) was obtained from GTEx portal (https://GTExportal.org/home/datasets).
