# Supplementary material for: Deep transcriptome sequencing of subgenual anterior cingulate cortex reveals cross-diagnostic and diagnosis-specific RNA expression changes in major psychiatric disorders
Source: Neuropsychopharmacology. 2021 Feb 8;46(7):1364–72. doi: 10.1038/s41386-020-00949-5 (PMC8134494; doi:10.1038/s41386-020-00949-5)
Supplement: Supplementary file 1 — Supplementary Methods [file 41386_2020_949_MOESM1_ESM.docx]

**METHODS**

**Samples:** All the 200 post-mortem brain samples (61 controls; 39 bipolar disorder; 46 schizophrenia; 54 major depression) were drawn from the National Institute of Mental Health (NIMH) Human Brain Collection Core (HBCC), collected by the Section on Neuropathology of the Clinical Brain Disorders Branch under protocol# 90-M-0142 [1]. All samples were dissected from frozen coronal slabs cut at autopsy. The portion of anterior cingulate cortex underlying the genu of the corpus callosum (generally including Brodmann areas 25, 32 and 24) was targeted. Dissections were done by dental drill cleaned between specimens (H2O/Bleach/H2O). Specimens were laid out on a cutting board placed on dry ice and kept frozen during dissections. Following dissection, total RNA was extracted from 50mg of pulverized subgenual anterior cingulate cortex. Samples with RIN <6 were excluded from sequencing.

**RNA sequencing**: Stranded RNA-Seq libraries were constructed from 1 µg total RNA after rRNA depletion using Ribo-Zero GOLD (Illumina Inc, San Diego, CA, USA). The Illumina TruSeq Stranded Total RNA Sample Prep Kit was used according to manufacturer’s instructions except where noted. Amplification was performed using 10 cycles which was optimized for the input amount and to minimize the chance of over-amplification. Samples were randomly distributed across libraries that were pooled together for sequencing in equimolar amounts. The pooled libraries were sequenced at the National Institute of Health Intramural Sequencing Center (NISC) on a HiSeq 2500 using version 4 chemistry. Stranded, paired-end sequencing data with read length of 125bp were processed using RTA version 1.18.64 and Casava 1.8.2.

**Mapping and counting:** Reads were trimmed using Trimmomatic [2] with default parameters (<http://www.usadellab.org/cms/?page=trimmomatic>). The trimmed reads were then mapped to the human genome (Ensembl GRCh38.87) using Hisat2 [3] (<https://ccb.jhu.edu/software/hisat2/index.shtml>). While 98% of the reads could be mapped to the human genome with default parameters, we used more stringent criteria that allowed only non-discordant and non-mixed reads, which resulted in ~60% of mapped reads. Known genes and transcripts mapped to autosomes and pseudo-autosomal regions (PAR) were included in the analysis. Gene and transcript counts were obtained using StringTie [3] software (<http://ccb.jhu.edu/software/stringtie/index.shtml?t=manual>).

**Sample selection:** Pre-noise filtered quantile normalized expression values for all 200 samples were used to calculate correlations between each possible pair of samples within a diagnostic category. The average correlation for each sample within each diagnostic category was obtained, providing a single estimate of relatedness of that sample to all others in its diagnostic category. We then tested for deviance of each sample to the rest of their diagnostic group by a z-score test, choosing a conservative threshold of p<0.1 to define individual samples as outliers. By this approach, 15 samples were identified and removed (Supplemental Figure 1), leaving a total of 185 samples (55 controls, 35 bipolar disorder, 44 schizophrenia, 51 major depression) for inclusion in the downstream analyses.

**Gene selection.** Known genes and transcripts with counts >0.1 TPM averaged across all 185 samples were included in the downstream analyses, comprising 21,228 Ensembl genes and 85,295 Ensembl transcripts.

**Covariate selection:** Since many factors can affect expression measurements in postmortem tissue, we employed a two-tiered strategy aimed at controlling for 1) variables for which diagnostic groups were substantially imbalanced, and 2) variables that were associated with major sources of variance in the gene expression data. We tested 32 known variables for association with diagnosis. These included RNA quality metrics (mapping percentage, GC percentage, 5-3’bias, RIN, extraction batch, library batch), available demographic data (age, gender, reported race, BMI, marital status, and manner of death), post-mortem interval (PMI), source of brain donor, tissue pH, and brain weight, along with post-mortem toxicology (the binary toxicology variables included were alcohol, opioids, major stimulants, hallucinogens, cocaine, sedative hypnotic anxiolitics, non psychiatric drugs, nicotine-cotinine, benzos, cannabinoids, antidepressants, anticholinergics, other psychotropic drugs, antipsychotics, mood stabilizers, anti-epileptics, and THC) results. In addition, 10 ancestry vectors were derived from genotyping on Illumina SNP arrays using Eigensoft [4, 5].

Stepwise logistic regression analysis was used to test association with the multinomial dependent variable of diagnostic group (bipolar, major depression, schizophrenia, control). Nominally significant associations were detected between diagnostic groups and race, source of brain donor, and antipsychotic exposure (Supplementary Table 3a). Of these, only antipsychotic exposure remained significant after Bonferroni-correction for the 43 covariates tested. Diagnostic groups were well-balanced in terms of the remaining variables.

Principal component (PC) analysis of the RNA counts data revealed that the top 10 PCs explained ~45% of the variance. Each of these PCs were tested for association with the known covariates by stepwise linear regression. Only race, RIN, and GC percent were significantly associated with one or more PCs at Bonferroni p<0.05 (Supplementary Table 3b). Note that the derived PCs were more strongly associated with reported race than with ancestry vectors.

On the basis of these results, downstream analyses of differential expression between diagnostic groups included race, RIN, and GC percent as covariates, while potential effects of antipsychotic exposure were tested in post-hoc analyses using approximate posterior estimation for GLM coefficients in DESeq2. In QTL analyses, which did not break down the sample by diagnosis and where ancestry-associated differences in linkage disequilibrium are important, 10 ancestry vectors were used along with RIN, race and GC percent.

**Sample and gender validation:**

Variant calling on RNA-seq data was carried out based on a modified GATK best practices pipeline. First, we used Picard (v2.20.8) (http://broadinstitute.github.io/picard/) to add read groups, sort and mark duplicates of the merged BAM files. Next, GATK (v4.1.3.0) [6] was used to split reads that contain Ns in their cigar string and carry out base quality recalibration. We used Ensembl variation data (Ensembl Release 97 database) as our reference database of known polymorphic sites. BCFtools (v1.9) [7] was used to call, filter, and annotate variants. Variants with QUAL score <20 were filtered out. Finally, we used PLINK (v1.9.0) [8] to process our VCFs and R to calculate concordance rates.

**Genotype data and Imputation:** Samples were genotyped on Illumina HumanHap650Y, Human1M-Duo, Omni5M-Quad-v1.0 and Omni5M-Quad-v1.1 arrays (Illumina Inc, San Diego, CA, USA). Markers that have minor allele frequency (MAF) <1%, genotype missing rate >5% and Hardy-Weingberg Equilibrium (HWE) p<10^-4^ were excluded. All individuals had >95% genotype rate (--mind 0.05). Approximately 300,000 common markers across the multiple arrays were extracted and files were merged using PLINK2.0 (<https://www.cog-genomics.org/plink/2.0/>). All the samples were imputed against the Haplotype Reference Consortium (HRC release 1.1 panel) and pre-phasing was done using EAGLE2 on Sanger imputation server [9] (<https://imputation.sanger.ac.uk>). After imputation any markers with R^2^ < 50%, MAF < 5%, and multi-allelic variants were excluded. All the markers were converted from hg19 to GRCh38 positions.

**Gene- and transcript-level analysis:** We normalized the raw counts using conditional quantile normalization [10] (CQN) (https://bioconductor.org/packages/release/bioc/html/cqn.html) which corrects for library size, gene length, and GC content. These normalized counts were then imported to DESeq2 [11] (<https://bioconductor.org/packages/release/bioc/html/DESeq2.html>) and transformed to variance stabilized data (VSD). As noted above, RIN, race and GC percent were significantly associated with diagnosis and RNA counts, so these were included as covariates in the downstream analysis. DESeq2 along with lfcShrink option was used for differential expression analysis between groups. The p-values were corrected for multiple testing using FDRtool [12] software in R. The significance of overlapping genes between groups is calculated using SuperExactTest in R.

**DAVID Functional annotation:** Functional annotation was performed in DAVID which is a database of annotation, visualization and integrated discovery [13] (https://david.ncifcrf.gov/content.jsp?file=citation.htm). All 85,295 transcripts that met criteria for inclusion in the DE analysis were used as background genes.

**Expression quantitative trait loci (eQTL) analysis:** Matrix eQTL [14] was used to identify eQTLs that were associated with expression at the gene or transcript levels (<http://www.bios.unc.edu/research/genomic_software/Matrix_eQTL/>). Variance stabilized data (VSD) from DESeq2 was used for this step, with RIN, race, GC percent, and 10 ancestry vectors included as covariates. Variants within 1MB of a gene were classified as “cis”. All default parameters were used. In the transcript-level analysis, transcripts with base mean<100 were excluded for computational efficiency.

**Combining eQTLs from different studies:** Perl scripts were used to remove any duplicate eQTLs and eQTLs that were on sex chromosomes. eQTLs that were common in sgACC, GTEx-ACC and CMC-DLPFC studies were combined using MeCS [15].

**SMR analysis:** Variants with pleiotropic effects on disease risk and RNA expression were identified by analyzing the eQTL data and GWAS data in SMR software [16] (<https://cnsgenomics.com/software/smr/#Overview>). Due to the complexity of the MHC region on chromosome6 (26MB-34MB), this region was excluded in the SMR analysis. All default parameters were used. Any markers with HEIDI P-value<0.05 were excluded.

**eQTL comparison with CMC-DFPLC and GTEx-ACC**: Since SNPs in the present study were mapped to hg38 while the Common Mind Consortium (CMC-DFPLC) and GTEx-ACC data were mapped to hg19, comparisons were based on rs-numbers rather than position. The CMC-DFPLC eQTL data was obtained through Synapse (https://www.synapse.org). GTEx-ACC data from anterior cingulate cortex (ACC) was downloaded from the GTEx website (<https://gtexportal.org/home/datasets>).

**Splicing quantitative trait loci (sQTL) analysis:** Variants associated with alternative splicing were identified by sQTLseekeR [17], an R package, with default parameters (<https://www.nature.com/articles/ncomms5698>). The sQTLs were characterized using AStalavista [18] software (<http://genome.crg.es/astalavista/>). Functional annotation of the sQTLs was performed using SNPnexus [19] (<http://www.snp-nexus.org>)

**Publicly available datasets:** The genome-wide association study (GWAS) results were obtained from PGC [20] (<http://www.med.unc.edu/pgc/results-and-downloads>) and the UKbiobank [21] (early access upon request). The Common Mind Consortium (CMC) eQTL dataset for the dorsolateral prefrontal cortex region (DFPLC) was downloaded from Synapse (<https://www.synapse.org>). The GTEx eQTL data for the anterior cingulate region (ACC) was obtained from the GTEx portal (https://GTExportal.org/home/datasets).

**Enrichment analysis:** For Schizophrenia, GWAS summary results, including index and credible SNPs, were downloaded from the PGC website (<https://www.med.unc.edu/pgc/results-and-downloads>). For Bipolar disorder and Major depression we extracted index SNPs with trait association p-values<10^-6^ then used PLINK [8] (http://zzz.bwh.harvard.edu/plink/) to extract all SNPs in linkage disequilibrium (r^2^>0.6) with one or more index SNPs (“LD friends”). We also used PLINK to calculate linkage disequilibrium values among the index SNPs, LD friends, and sQTLs (p<0.05). We then extracted sQTL-GWAS SNP pairs with r^2^>0.6. The pairs were then pruned to get independent loci using the “indep-pairwise” option in PLINK (--indep-pairwise 50 5 0.5). The 1000Genomes European data[22] were used as reference (<http://www.internationalgenome.org/data>).

**Heritability estimates:** Heritability estimates were performed with LDSC [23] (<https://github.com/bulik/ldsc>), using the GWAS summary statistics for schizophrenia, bipolar disorder, and MDD (noted above). For this analysis, Alzheimer Disease was used as a negative control, based on GWAS summary statistics downloaded from IGAP (<http://web.pasteur-lille.fr/en/recherche/u744/igap/igap_download.php>) [24].

In order to increase the power, sQTLs and transcript eQTLs from our study were combined with PsychENCODE isoQTLs and tQTLs, respectively [25]. To perform the partitioned heritability analysis for sQTLs and eQTLs we created a custom annotation file, designating each SNP as an sQTL or an eQTL that was not an sQTL according to the instructions given in LDSC software (<https://github.com/bulik/ldsc/wiki/LD-Score-Estimation-Tutorial>). This annotation file was then used to partition the heritability between sQTLs and eQTLs in each disorder.

**qPCR validation**

Some genes and transcripts with expression that was high enough to produce reliable results by qPCR (baseMean > 500) and that were identified as DE in the DESeq2 analysis of the RNA-seq data were selected for validation. At the gene-level, we selected *DUSP1* and *NR4A2*, both of which were DE in BD, and *HIGD2A*, which was DE in BD and MDD. At the transcript level, we selected transcripts of *ARID5B* (ENST00000279873), *NT5DC3* (ENST00000392876) and *SF3A1* (ENST00000215793) that were DE in SCZ. Taqman probes were ordered from Applied Biosystems (Thermo Fisher Scientific, 168 Third Avenue, Waltham, MA, USA). Catalog numbers are Hs00610256_g1, Hs00431157_g1, Hs01117527_g1, Hs01381961_m1, Hs00213132_m1, and Hs01066327_m1.

**Methods References**

1. Lipska BK, Deep-Soboslay A, Weickert CS, Hyde TM, Martin CE, Herman MM, et al. Critical Factors in Gene Expression in Postmortem Human Brain: Focus on Studies in Schizophrenia. Biol Psychiatry. 2006;60:650–658.

2. Bolger AM, Lohse M, Usadel B. Trimmomatic: a flexible trimmer for Illumina sequence data. Bioinformatics. 2014;30:2114–2120.

3. Pertea M, Kim D, Pertea GM, Leek JT, Salzberg SL. Transcript-level expression analysis of RNA-seq experiments with HISAT, StringTie and Ballgown. Nat Protoc. 2016;11:1650–1667.

4. Galinsky KJ, Bhatia G, Loh P-R, Georgiev S, Mukherjee S, Patterson NJ, et al. Fast Principal-Component Analysis Reveals Convergent Evolution of ADH1B in Europe and East Asia. Am J Hum Genet. 2016;98:456–472.

5. Galinsky KJ, Loh P-R, Mallick S, Patterson NJ, Price AL. Population Structure of UK Biobank and Ancient Eurasians Reveals Adaptation at Genes Influencing Blood Pressure. Am J Hum Genet. 2016;99:1130–1139.

6. McKenna A, Hanna M, Banks E, Sivachenko A, Cibulskis K, Kernytsky A, et al. The Genome Analysis Toolkit: a MapReduce framework for analyzing next-generation DNA sequencing data. Genome Res. 2010;20:1297–1303.

7. Li H. A statistical framework for SNP calling, mutation discovery, association mapping and population genetical parameter estimation from sequencing data. Bioinforma Oxf Engl. 2011;27:2987–2993.

8. Purcell S, Neale B, Todd-Brown K, Thomas L, Ferreira MAR, Bender D, et al. PLINK: a tool set for whole-genome association and population-based linkage analyses. Am J Hum Genet. 2007;81:559–575.

9. McCarthy S, Das S, Kretzschmar W, Delaneau O, Wood AR, Teumer A, et al. A reference panel of 64,976 haplotypes for genotype imputation. Nat Genet. 2016;48:1279–1283.

10. Hansen KD, Irizarry RA, WU Z. Removing technical variability in RNA-seq data using conditional quantile normalization. Biostat Oxf Engl. 2012;13:204–216.

11. Love MI, Huber W, Anders S. Moderated estimation of fold change and dispersion for RNA-seq data with DESeq2. Genome Biol. 2014;15:550.

12. Strimmer K. fdrtool: a versatile R package for estimating local and tail area-based false discovery rates. Bioinformatics. 2008;24:1461–1462.

13. Huang DW, Sherman BT, Lempicki RA. Systematic and integrative analysis of large gene lists using DAVID bioinformatics resources. Nat Protoc. 2009;4:44–57.

14. Shabalin AA. Matrix eQTL: ultra fast eQTL analysis via large matrix operations. Bioinformatics. 2012;28:1353–1358.

15. Qi T, Wu Y, Zeng J, Zhang F, Xue A, Jiang L, et al. Identifying gene targets for brain-related traits using transcriptomic and methylomic data from blood. Nat Commun. 2018;9:2282.

16. Zhu Z, Zhang F, Hu H, Bakshi A, Robinson MR, Powell JE, et al. Integration of summary data from GWAS and eQTL studies predicts complex trait gene targets. Nat Genet. 2016;48:481–487.

17. Monlong J, Calvo M, Ferreira PG, Guigó R. Identification of genetic variants associated with alternative splicing using sQTLseekeR. Nat Commun. 2014;5:4698.

18. Foissac S, Sammeth M. ASTALAVISTA: dynamic and flexible analysis of alternative splicing events in custom gene datasets. Nucleic Acids Res. 2007;35:W297–W299.

19. Dayem Ullah AZ, Oscanoa J, Wang J, Nagano A, Lemoine NR, Chelala C. SNPnexus: assessing the functional relevance of genetic variation to facilitate the promise of precision medicine. Nucleic Acids Res. 2018;46:W109–W113.

20. Stahl E, Breen G, Forstner A, McQuillin A, Ripke S, Consortium BDWG of the PG, et al. Genomewide association study identifies 30 loci associated with bipolar disorder. BioRxiv. 2018:173062.

21. Howard DM, Adams MJ, Shirali M, Clarke T-K, Marioni RE, Davies G, et al. Genome-wide association study of depression phenotypes in UK Biobank identifies variants in excitatory synaptic pathways. Nat Commun. 2018;9:1470.

22. The 1000 Genomes Project Consortium. A global reference for human genetic variation. Nature. 2015;526:68–74.

23. Zheng J, Erzurumluoglu AM, Elsworth BL, Kemp JP, Howe L, Haycock PC, et al. LD Hub: a centralized database and web interface to perform LD score regression that maximizes the potential of summary level GWAS data for SNP heritability and genetic correlation analysis. Bioinforma Oxf Engl. 2017;33:272–279.

24. Lambert JC, Ibrahim-Verbaas CA, Harold D, Naj AC, Sims R, Bellenguez C, et al. Meta-analysis of 74,046 individuals identifies 11 new susceptibility loci for Alzheimer’s disease. Nat Genet. 2013;45:1452–1458.

25. Gandal MJ, Zhang P, Hadjimichael E, Walker RL, Chen C, Liu S, et al. Transcriptome-wide isoform-level dysregulation in ASD, schizophrenia, and bipolar disorder. Science. 2018;362:eaat8127.
