# Supplementary material for: Deep transcriptome sequencing of subgenual anterior cingulate cortex reveals cross-diagnostic and diagnosis-specific RNA expression changes in major psychiatric disorders
Source: Neuropsychopharmacology. 2021 Feb 8;46(7):1364–72. doi: 10.1038/s41386-020-00949-5 (PMC8134494; doi:10.1038/s41386-020-00949-5)
Supplement: Supplementary file 2 — Supplementary Figures [file 41386_2020_949_MOESM2_ESM.docx]

Supplemental Figure 1: Pearson correlation matrix before and after removal of outlier samples. The color bar indicates Pearson correlation.


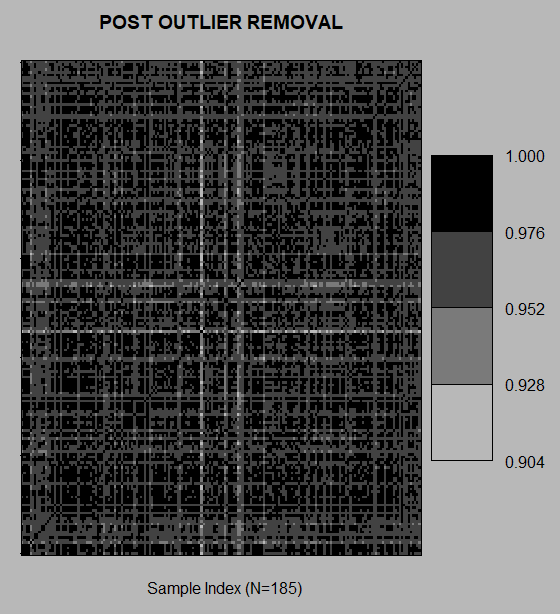

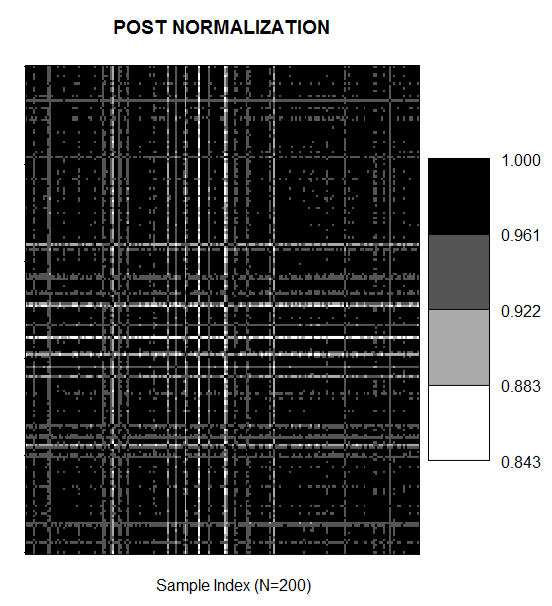

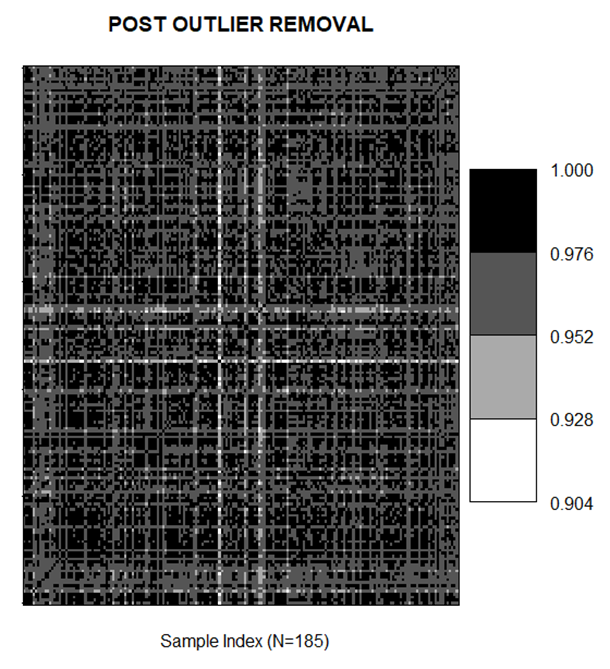


Supplemental Figure 2: Analysis pipeline of subgenual anterior cingulate cortex (sgACC) transcriptome

Supplemental Figure 3: Log_2_ fold change comparison of differentially expressed (DE) genes and transcripts in RNA sequencing and qPCR.

Supplemental Figure 4. Volcano plots of differentially expressed genes and transcripts in case control comparisons. Log_2_ fold-change values are plotted against the X-axis and -log_10_ p-values for differential expression are shown on the Y-axis. Genes with FDR<5% are highlighted in red. Gene-level results are shown for a. case-control and b. case-case comparisons; corresponding transcript-level results are shown in panels c. and d., respectively. Note that for case-case comparisons the sign of the log_2_ fold-change is arbitrary.

Case-control

Genes

Transcripts

Case-case Case-control


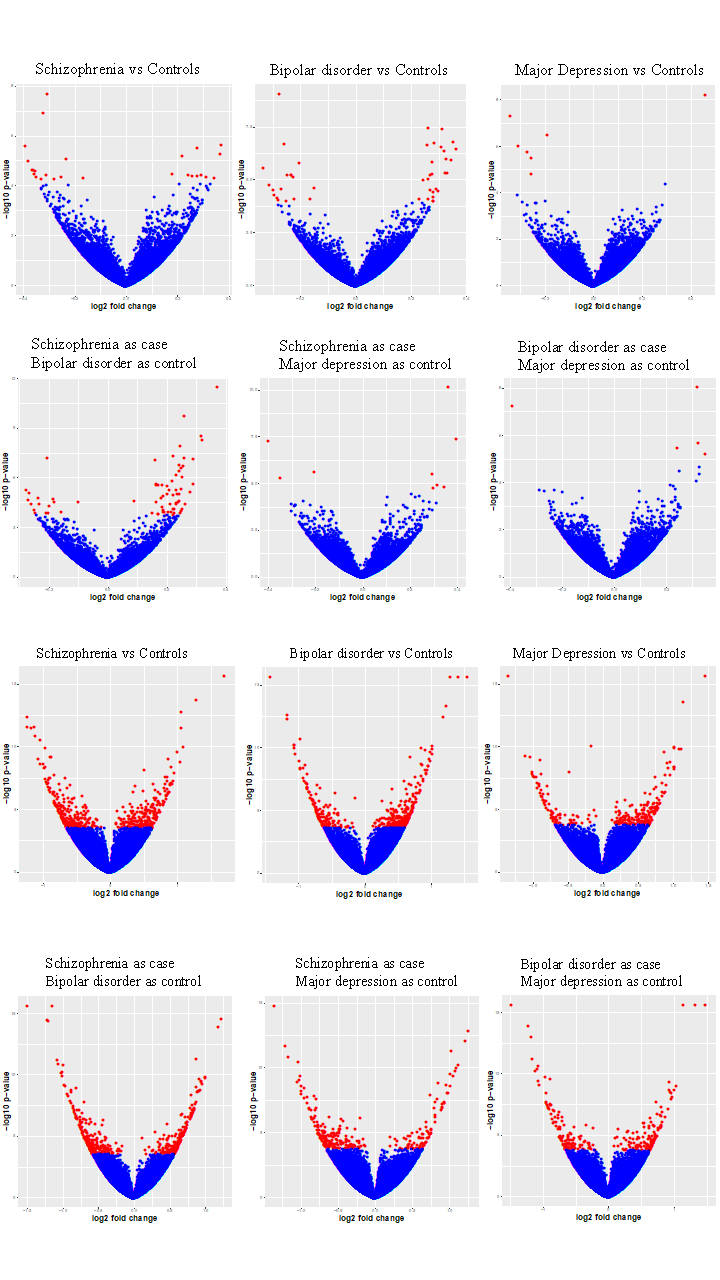

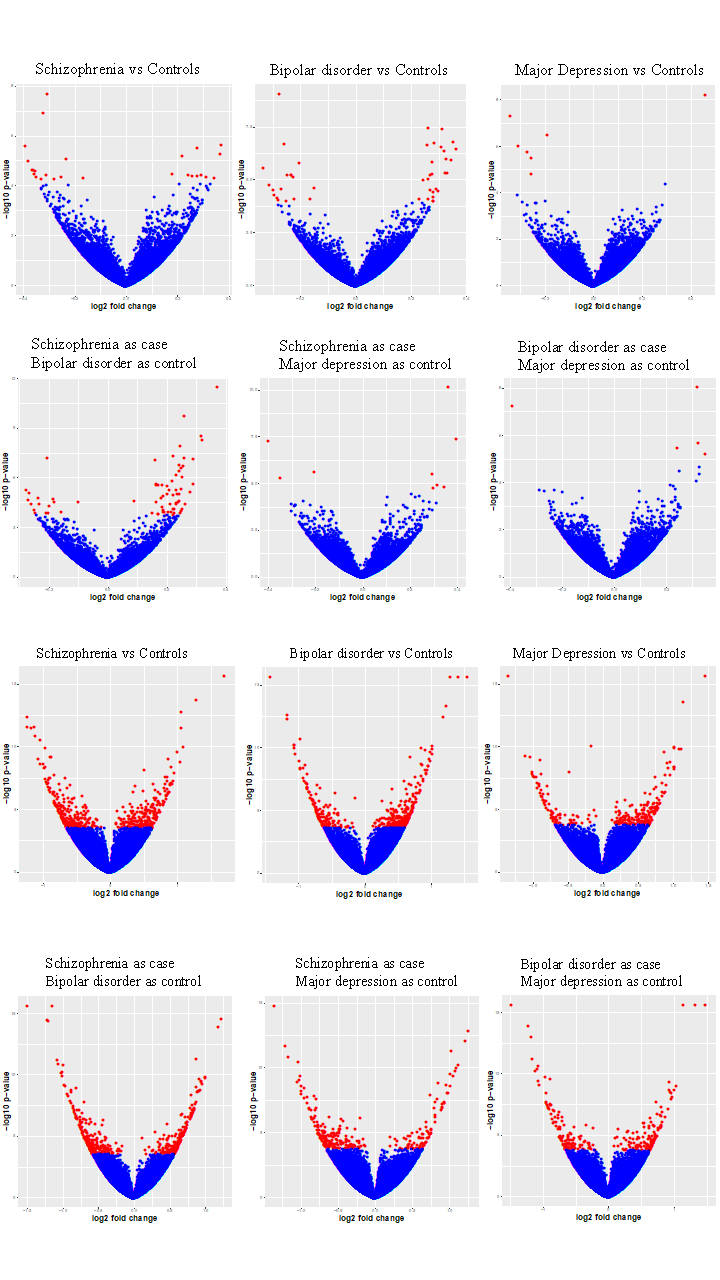

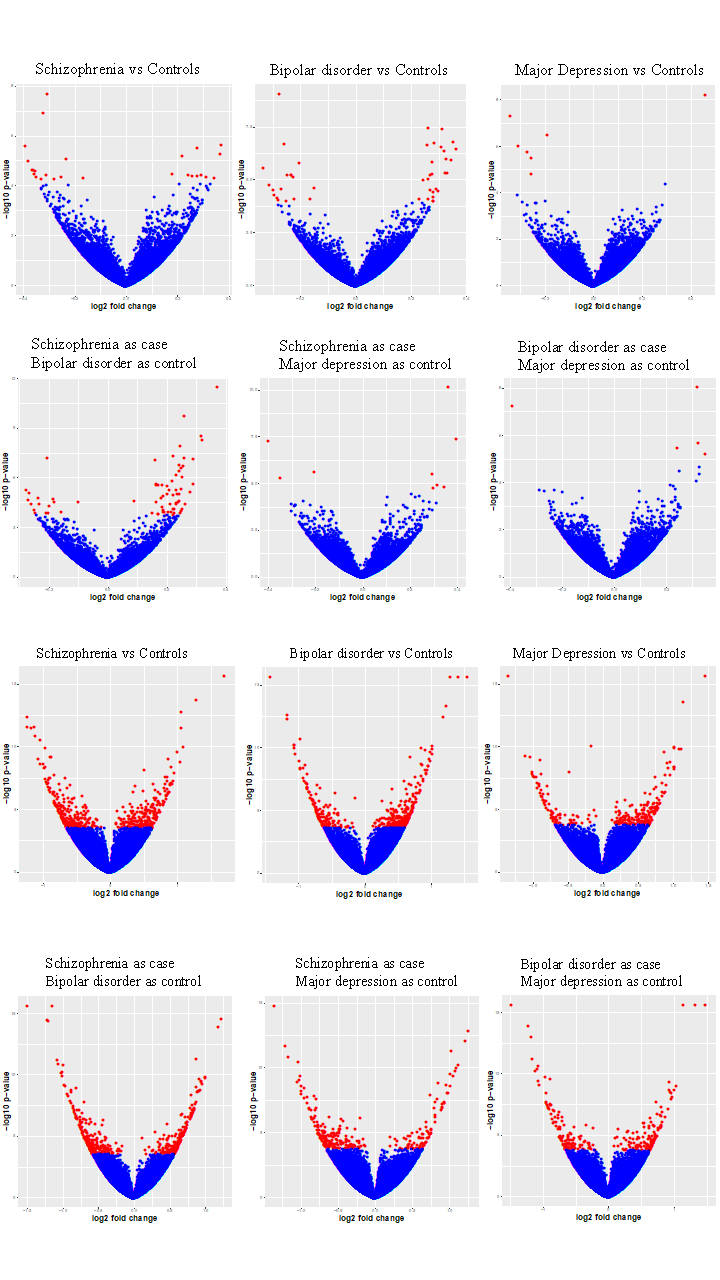


a

b

c

d

Supplemental Figure 5: eGenes (FDR<5%) overlap between subgenual anterior cingulate cortex (sgACC), Common Mind Consortium (CMC) and GTex anterior cingulate cortex (GTexACC).


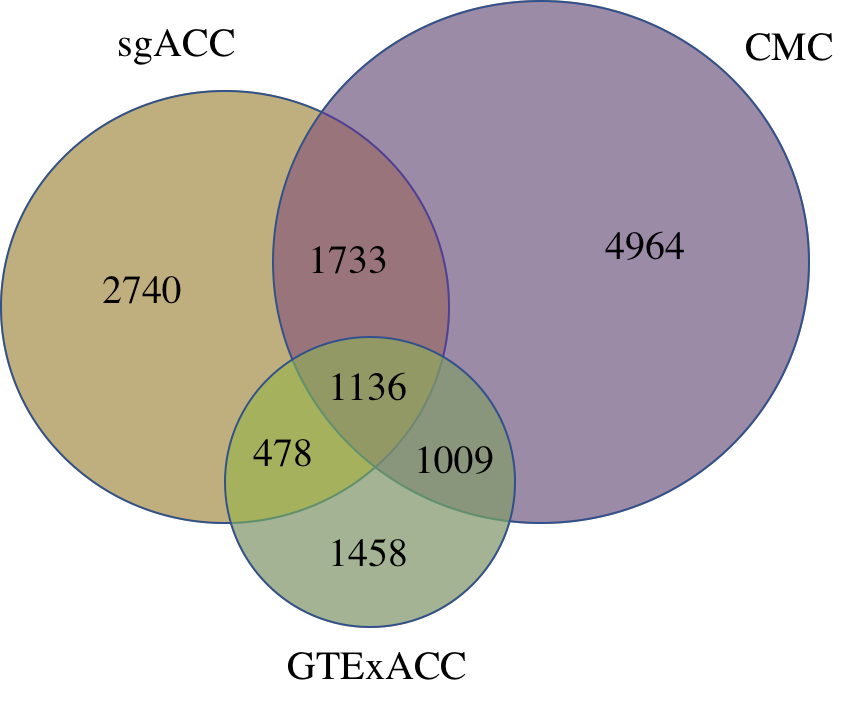


Supplemental Figure 6: Read count distribution of transcripts differentially expressed in any disorder (FDR<5%)

Supplemental Figure 7: **Multiple transcripts of the same genes differentially-expressed across disorders.** Genes with multiple differentially-expressed transcripts in bipolar disorder (BD), schizophrenia (SCZ), or major depressive disorder (MDD). Within each diagnosis column, downregulated transcripts are shown in red to the left, under “-“, upregulated transcripts are shown in blue to the right, under “+”. Across each row, different shapes indicate distinct transcripts. Full gene and transcript names are shown in eTables 16-18 in the Supplement.


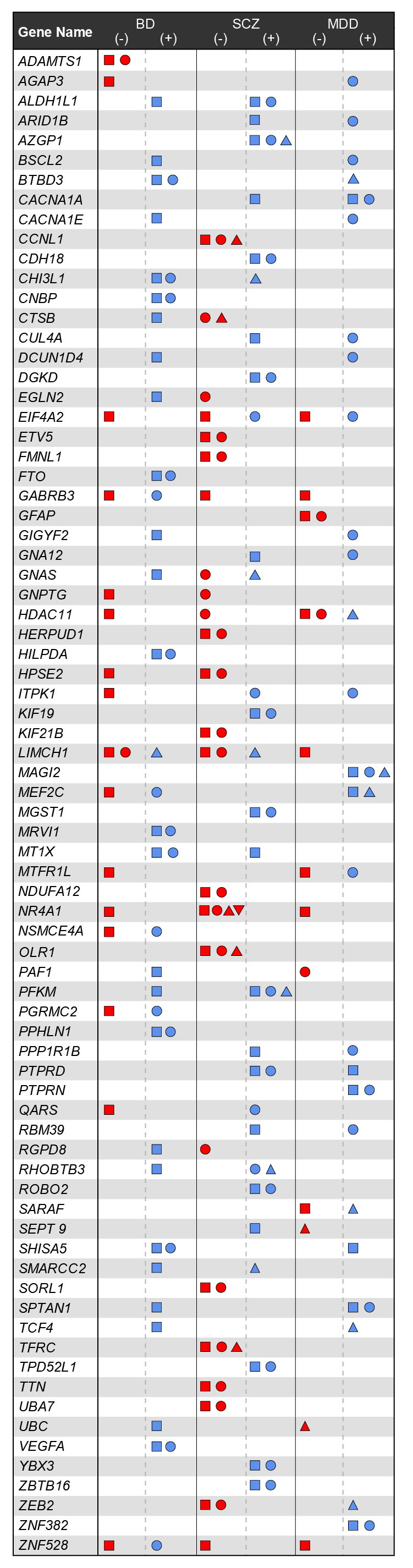


Supplemental Figure 8: Box plots of sQTL effect on relative transcript abundance for the top 3 genes in eTable 32 (in the Supplement) are presented below. The two transcripts with the largest genotype effects (as identified by sQTLseekeR) are shown for each gene. Genotypes are shown on the x-axis and the relative transcript abundance is shown on the y-axis.


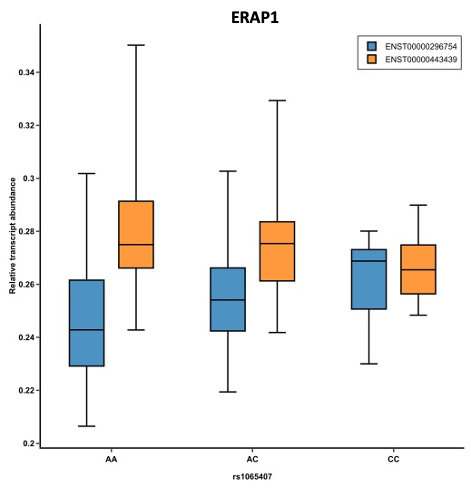

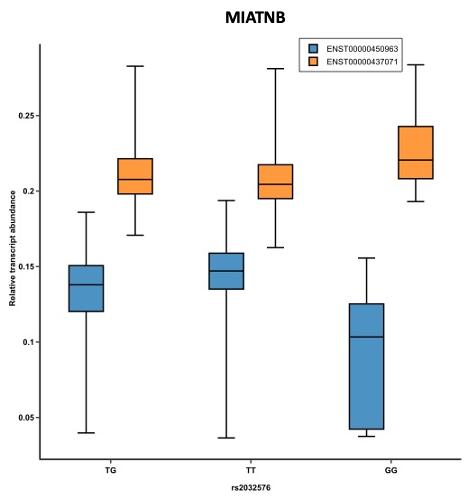

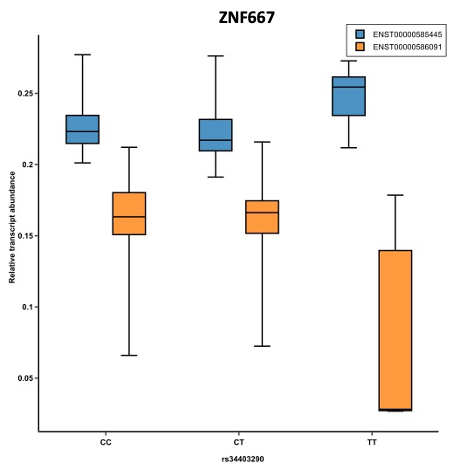


Supplemental Figure 9: Regulatory annotation of sQTLs. ENCODE annotation terms are shown on the y-axis, total counts of identified sQTLs (p<0.05) are shown on the x-axis.


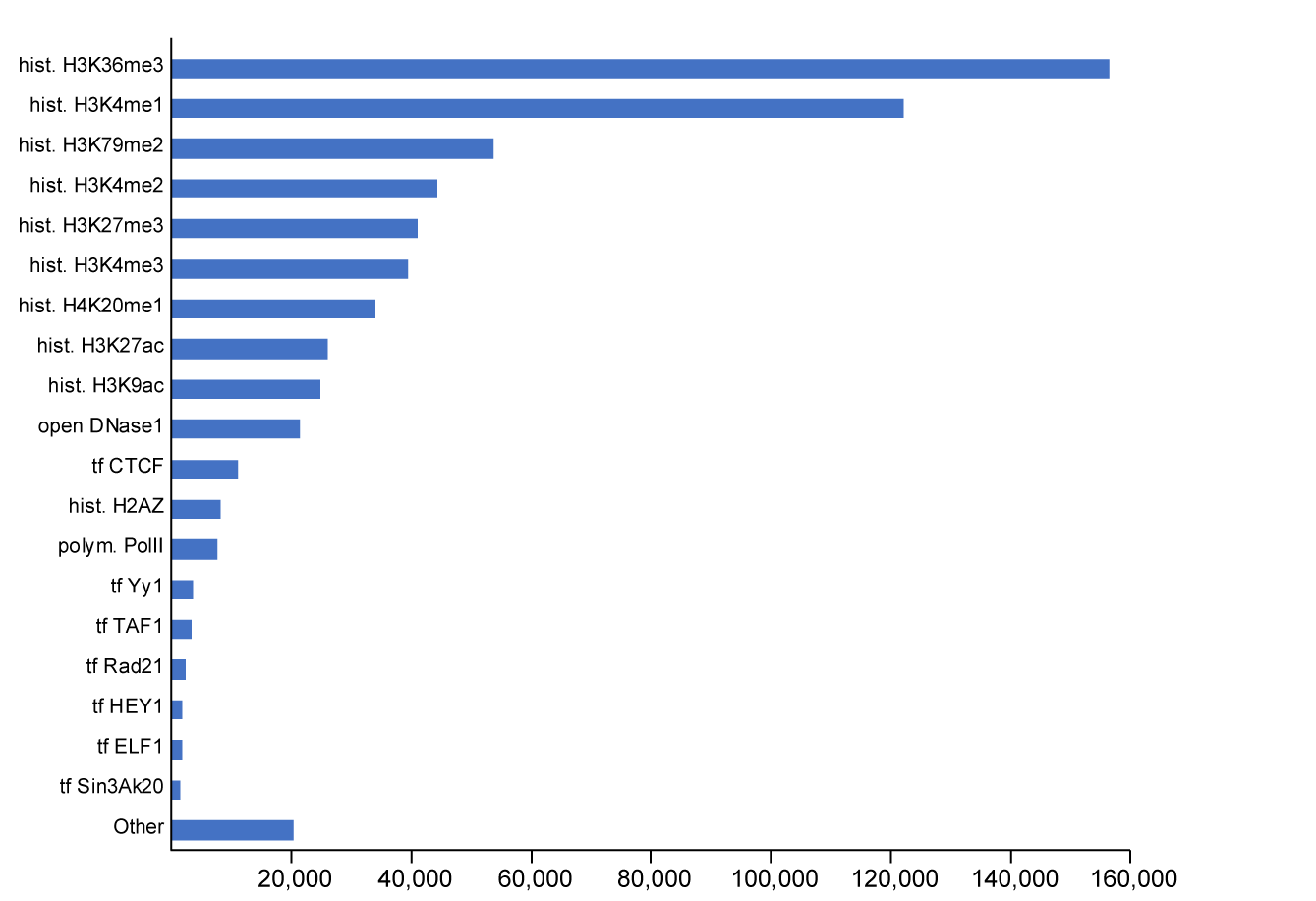


Supplemental Figure 10: Histogram of distances between sQTLs (p<0.05, Caucasian-only) and nearest splicing site

Supplemental Figure 11: Breakdown of predicted alternative splicing events by type for all sQTLs (Caucasian only, p<0.05)
